# Physical activity maintenance and increase in Chinese children and adolescents: the role of intrinsic motivation and parental support

**DOI:** 10.3389/fpubh.2023.1175439

**Published:** 2023-07-31

**Authors:** Yujie Liu, Xin Ge, Huilun Li, Erliang Zhang, Fan Hu, Yong Cai, Mi Xiang

**Affiliations:** School of Public Health, Shanghai Jiao Tong University, Shanghai, China

**Keywords:** children and adolescents, physical activity, intrinsic motivation, parental support, parental physical activty, longitudinal study

## Abstract

**Objective:**

This longitudinal study aimed to examine the association of intrinsic motivation, parental physical activity, and parental support with physical activity maintenance and increase among children and adolescents.

**Methods:**

A sample of 2,424 children and adolescents in Shanghai, China participated in the two-wave survey before and during the COVID-19 pandemic. The questionnaire measured children and adolescents’ physical activity and intrinsic motivation, as well as their parental physical activity and support (concern for their child and co-activity with their child). Multivariable logistic regressions were performed by groups to examine the associations between these factors and physical activity change.

**Results:**

Most children and adolescents exhibited a decline in physical activity participation during the pandemic, as indicated by a mere 15.0 and 8.0% of individuals maintaining and increasing their pre-pandemic levels, respectively. Among the initially active participants, perceived self-choice [OR = 1.341 (95%CI: 1.173–1.533)] and parental concern [OR = 1.922 (95%CI: 1.204–3.068)] predicted maintained physical activity. Increased physical activity was predicted by perceived enjoyment [OR = 1.193 (95%CI: 1.046–1.362)] and parental co-activity (OR = 1.995 [95%CI: 1.095–3.633]).

**Conclusion:**

This study provides longitudinal evidence that intrinsic motivation and parental support can have a positive impact when physical activity levels change significantly. Effective interventions targeting multilevel factors are needed to maintain or increase children and adolescents’ physical activity.

## 1. Introduction

Physical activities present great health benefits to children and adolescents, including maintaining a healthy weight, improving cardiometabolic health, and ensuring mental well-being and physical fitness (1). However, current physical activity levels of adolescents remain low, with 80% of global adolescents being insufficiently active to achieve the recommended 60 min of moderate-to-vigorous physical activity per day (2). Moreover, the physical activity level of children and adolescents does not remain constant but declines significantly with age (3). The declining trend tends to proceed in later adulthood and has a long-lasting influence on health status throughout life (4). Longitudinal studies have confirmed the favorable effects of maintaining or increasing physical activities on cardiovascular disease risks and mental health status (5). Therefore, physically inactive children and adolescents may increase their risk of several diseases, which is recognized as a major public health problem, with interventions warranted to promote regular physical activity participation in this population (6).

Understanding the psychological mechanism underlying physical activity behavior is pivotal to implementing effective physical activity intervention in children and adolescents. Self-determination theory (SDT) is a behavioral change theory that has been successfully used for both explaining the motivational dynamics of physical activity behaviors and developing theory-grounded physical activity intervention programs in children and adolescents (7, 8). SDT focuses primarily on intrinsic motivation that innately drives people’s behaviors (e.g., engaging in an activity that is naturally appealing) (9). Intrinsic motivation has been recognized as a significant contributor to physical activity participation in children and adolescents by numerous studies (8). For example, Nogg et al. found that intrinsic motivation was positively associated with physical activity across different settings (in and out of school) in U.S. adolescents (10).

Beyond personal motivational factors, environmental support of basic psychological needs for autonomy, competence, and relatedness is another key component of SDT, influencing one’s behavior *via* fostering or thwarting motivation (11). During childhood and adolescence, physical activity changes rely heavily on the involvement of family members (12). Parental support, an omnibus of various support behaviors for physical activity engagement, is essential in promoting physical activity among children and adolescents (13, 14). In some cases, parental support for physical activity can be grouped into tangible and intangible forms of support (15). Empirical evidence suggests that both tangible support (e.g., providing transportation or engaging in exercise with their child) and intangible support (e.g., providing encouragement or care in exercise) have been associated with increased levels of children’s physical activity (15–18). In addition to the facilitation of child physical activity, parental engagement in physical activity is another critical determinant of activity-related behaviors in young people, which may affect physical activity through various mechanisms, such as role modeling (19). Findings from a review demonstrate a weak positive association between parental and children’s physical activity levels (20). However, results regarding this association are inconsistent and lack longitudinal evidence (21). Further investigation is needed to elucidate the impacts of parental behaviors on children and adolescents’ physical activity participation.

**Figure 1:**
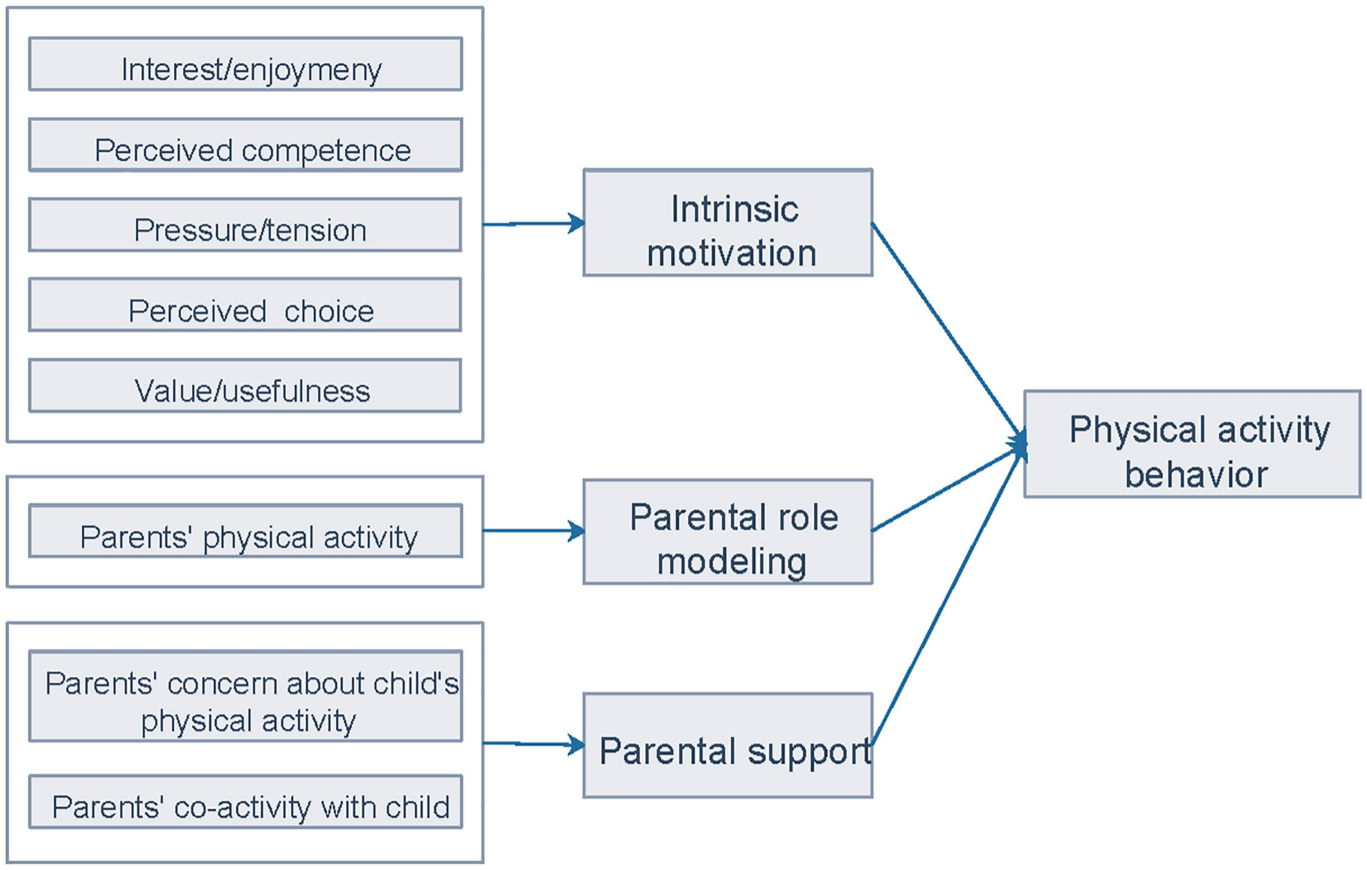
Conceptual model of SDT-related factors and physical activity behavior.

The hypothesized model of SDT-related factors and physical activity behavior is presented in Figure 1. Despite the salience of these factors on physical activity, few studies have explored how the individual and parental elements simultaneously impact children and adolescents’ physical activity over time. Furthermore, longitudinal studies of SDT-related factors have mainly focused on the long-term trend in physical activity. However, children and adolescents tend to experience a short-term dramatic decrease in physical activity due to major life events (22). The present study, which was conducted in the context of the coronavirus disease 2019 (COVID-19) pandemic, was able to capture significant predictors for maintaining or increasing physical activity when experiencing major changes in the social environment. During this special period, home confinement has reduced overall access to physical activity, resulting in dramatic lifestyle changes among children and adolescents (23). An investigation into the determinants of short-term physical activity change can offer valuable insights for preventive measures in the event of the recurrence of similar circumstances, to mitigate their potential negative impacts. Therefore, the aim of this study was to examine the associations of intrinsic motivation, parental physical activity, and parental support with short-term physical activity maintenance and increase before and during COVID-19.

## 2. Materials and methods

### 2.1. Participants

This web-based longitudinal study used multi-stage cluster sampling to recruit participants. Of the 14 districts of Shanghai that were invited, seven agreed to participate in the survey. In each of the seven districts, 1 to 2 schools were randomly selected. A total of ten schools participated in the baseline survey, and five of them participated in the follow-up surveys. There was no significant difference in sociodemographic variables between the retained five schools and the excluded five schools. All the children and adolescents in the selected schools and their parents were invited.

**Figure 2:**
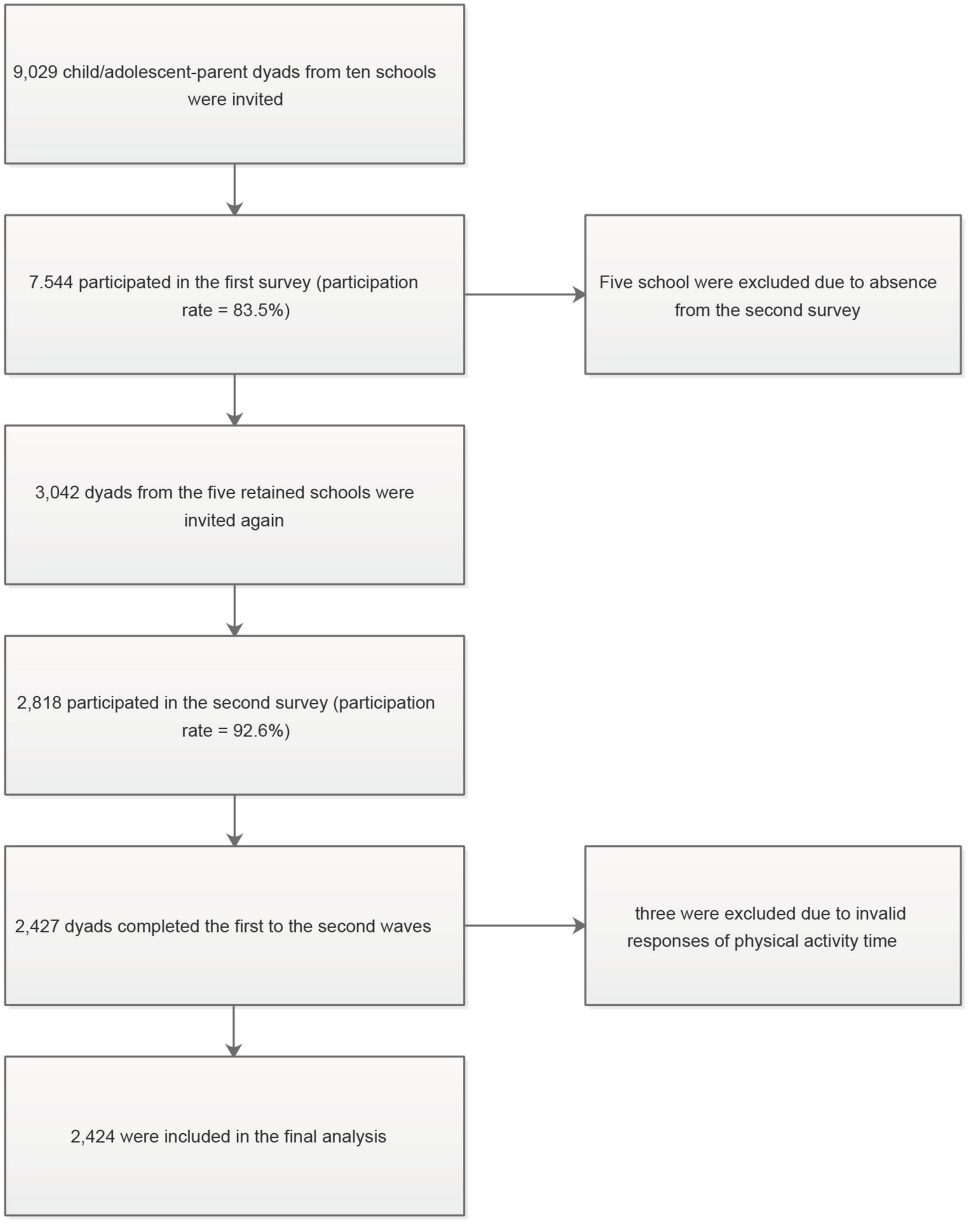
Flow of participants.

The first survey was conducted among 7,544 children and adolescents in the ten schools as well as their parents (approximately 83% participation rate) between January 3^rd^ and 21^st^, 2020. A Level 1 public health emergency was subsequently declared in Shanghai on January 24th, 2020. The lockdown began and children and adolescents were restricted to stay at home during this period. Approximately two months after the COVID-19 outbreak, the second survey was conducted among 2,818 children, adolescents, and their parents in the five schools (approximately 92% participation rate) between March 13^th^ and 23^rd^, 2020, right before the public health emergency response downgraded to Level 2 and the strict lockdown was relaxed. A total of 2,427 students and parents completed the first to the second waves. Participants with invalid responses to physical activity time (> 24 h per day) were excluded. The final sample consisted of 2,424 children and adolescents. The flow chart of participants in this study is presented in Figure 2.

In each survey wave, we first distributed information about the study among school head teachers and obtained their permission. The questionnaire was then distributed online by the head teacher to each parent and their children. Written informed consent from each parent was obtained before data collection. The parents and their children were asked to complete two separate parts of the questionnaire. The parents were instructed to assist primary school students in the process.

### 2.2. Measures

The questionnaire consisted of a child/adolescent section and a parent section. Physical activity and intrinsic motivation were derived from the child/adolescent section, while parental physical activity and parental support were derived from the parent section.

#### 2.2.1. Physical activity

Physical activities were assessed by the Global Physical Activity Questionnaire (GPAQ), which was developed by the World Health Organization (WHO) (24) and modified for use in children and adolescents in previous studies (25, 26). The GPAQ consists of 16 questions measuring physical activity participation in different settings. A person’s total time of physical activity participation in a week was then calculated using the GPAQ data. According to the WHO guidelines on physical activity for children and adolescents, active was defined as at least an average of 60 min per day of moderate-to vigorous-intensity physical activity across the week, and inactive was defined as less than an average of 60 min per day (1). In this study, physical activity increase was defined as a transition from a state of inactive to active; physical activity maintenance was defined as a sustained state of being active.

#### 2.2.2. Intrinsic motivation

Children and adolescents’ intrinsic motivation was assessed by five statements derived from five subscales of the Intrinsic Motivation Inventory (IMI) (27). The statements included (1) I enjoyed physical activities very much (interest/enjoyment); (2) I think I am pretty good at physical activities (perceived competence); (3) I did not feel nervous at all while doing physical activities (pressure/tension); (4) I felt like it was not my own choice to do physical activities (perceived choice); and (5) I believe these activities could be of some value to me (value/usefulness). Participants were asked to self-rate how each statement was true for them using a score ranging from 1 (not at all true) to 7 (very true). All of the responses are positively scored except for the one regarding “pressure/tension.”

#### 2.2.3. Parental physical activity

The GPAQ, which was originally designed for the adult population (28), was also completed by parents to assess their physical activity participation. The parent version of the GPAQ has the same item format and response as the child/adolescent version, except for the settings where physical activity participation was measured. Parental physical activity was presented as the total hours spent in physical activity in a week.

#### 2.2.4. Parental support

Parental support included two aspects of intangible support (concern about their child’s physical activity) and tangible support (co-activity with their child), respectively. Parental concern was assessed by a single question “How much do you concern about your child’s physical activity in your current life?.” Possible responses included “occasionally care,” “frequently care,” and “always care.” Parental co-activity was assessed by another question “How often do you engage in physical activity with your child?.” Possible responses included “Never,” “1 ~ 4 times per month,” and “>1 time per week.”

#### 2.2.5. Demographic covariates

Sex (male; female), age, and grade (1–3; 4–6; 7–9) were reported by children and adolescents. Mother’s and father’s educational level (junior high school and below; senior high school; college/university; master/doctor) and family annual income (<100,000¥, 100,000-200,000¥, 200,000-400,000¥, and > 400,000¥) were reported by parents.

### 2.3. Statistical analysis

Descriptive analyses were conducted for the baseline physical activity level on sample characteristics. Categorical variables were described by numbers and frequencies. Age was described by mean and standard deviation (SD), while IMI scores and parents’ physical activity were described using median and Q1-Q3 quartiles. Comparisons of all the baseline variables between the active group and inactive group were conducted using the Chi-square tests for categorical variables and the Wilcoxon rank-sum tests for continuous variables.

Participants were further categorized into active and inactive groups according to their assessments of physical activity level at follow-up. A series of logistic regressions were performed separately in the initially active and inactive participants, and odds ratios (with 95% confidence intervals) were calculated to compare the active and inactive participants at follow-up. Participants with missing data on parental physical activity, parental concern of their children’s active level, and parental frequency of coactivity with their child (n = 122) were excluded from the analyses. There were no significant differences in the sex or age distribution between the included and excluded participants (Supplementary Table S1). The bivariable associations between each factor and physical activity were examined using an unadjusted model and a multivariable model adjusting for demographic factors. The final multivariable model included variables with a value of p of less than 0.1 using the forward stepwise method considering the high collinearity among candidate predictors. All statistical analyses were performed using R 3.6.1. A value of *p* less than 0.05 was considered significant.

### 2.4. Ethics statement

The study was approved by the Ethics Committee of Shanghai Jiaotong University School of Medicine (SJUPN– 201813). Written informed consent was obtained from all the parents.

## 3. Results

### 3.1. Baseline sample characteristics

Of the included 2,424 children and adolescents, 1,241 were boys (51.2%) and 1,183 (48.8%) were girls, ranging in age from 6 to 15 [mean age (SD): 11.1 (2.67) years]. At baseline, 1,120 (46.2%) children and adolescents were physically active. Descriptive statistics of baseline sample characteristics according to the physical activity level are presented in Table 1.

**Table 1:** Baseline sample characteristics according to physical activity level.

Characteristics	Total (*n* = 2,424)	Inactive (*n* = 1,304)	Active (*n* = 1,120)	*p* value
Sex				
Male	1,241 (51.2%)	620 (47.5%)	621 (55.4%)	<0.001
Female	1,183 (48.8%)	684 (52.5%)	499 (44.6%)	
Age	11.1 ± 2.67	10.8 ± 2.74	11.4 ± 2.54	<0.001
Grade				
1–3	573 (23.6%)	349 (26.8%)	224 (20.0%)	<0.001
4–6	858 (35.4%)	472 (36.2%)	386 (34.5%)	
7–9	993 (41.0%)	483 (37.0%)	510 (45.5%)	
Mother education				
≤ Junior high school	226 (9.3%)	111 (8.5%)	115 (10.3%)	0.724
Senior high school	445 (18.4%)	238 (18.3%)	207 (18.5%)	
College/University	1,496 (61.7%)	819 (62.8%)	677 (60.4%)	
Master/Doctor	119 (4.9%)	63 (4.8%)	56 (5.0%)	
Unknown	138 (5.7%)	73 (5.6%)	65 (5.8%)	
Father education				
≤ Junior high school	170 (7.0%)	72 (5.5%)	98 (8.8%)	0.017
Senior high school	521 (21.5%)	274 (21.0%)	247 (22.1%)	
College/University	1,385 (57.1%)	773 (59.3%)	612 (54.6%)	
Master/Doctor	209 (8.6%)	108 (8.3%)	101 (9.0%)	
Unknown	139 (5.7%)	77 (5.9%)	62 (5.5%)	
Family income				
<100,000	276 (11.4%)	147 (11.3%)	129 (11.5%)	0.013
100,000-200,000	677 (27.9%)	371 (28.5%)	306 (27.3%)	
200,000-400,000	766 (31.6%)	430 (33.0%)	336 (30.0%)	
>400,000	376 (15.5%)	208 (16.0%)	168 (15.0%)	
Unknown	329 (13.6%)	148 (11.3%)	181 (16.2%)	
Intrinsic motivation				
Interest/Enjoyment	5 (4–7)	5 (3–6)	6 (4–7)	<0.001
Perceived Competence	6 (4–7)	5 (4–7)	6 (5–7)	<0.001
Pressure/Tension	2 (1–3)	2 (1–4)	1 (1–3)	<0.001
Perceived Choice	6 (4–7)	5 (3–7)	6 (5–7)	<0.001
Value/Usefulness	7 (6–7)	7 (5–7)	7 (6–7)	<0.001
Parental physical activity^*^	2.50 (0.08–7.00)	2.00 (0.00–5.83)	3.33 (1.00–8.50)	<0.001
Parental concern^*^				
Occasionally	772 (33.5%)	472 (38.1%)	300 (28.2%)	<0.001
Frequently	776 (33.7%)	397 (32.0%)	379 (35.7%)	
Always	754 (32.8%)	370 (29.9%)	384 (36.1%)	
Parental coactivity^*^				
Never	597 (25.9%)	347 (28.0%)	250 (23.5%)	<0.001
1 ~ 4 times per month	1,034 (44.9%)	575 (46.4%)	459 (43.2%)	
>1 time per week	671 (29.1%)	317 (25.6%)	354 (33.3%)	

Data are presented as mean ± standard deviation or median (Q1—Q3 quartiles) for continuous variables and *n* (%) for categorical variable.

^*^Participants with missing data on parental physical activity, parental level of concern about their child’s physical activity, and parental frequency of coactivity with their child (*n* = 122) were excluded from analyses.

The results of chi-square tests showed that sex (*p* < 0.001), age (p < 0.001), grade (p < 0.001), father’s educational level (*p* = 0.017), and annual family income (*p* = 0.014) were associated with physical activity level. Compared to those in the inactive group, the active children and adolescents scored higher on all the items of intrinsic motivation (*p* < 0.001). In addition, all the parental factors (*p* < 0.001) were positively associated with children and adolescents’ physical activity levels.

Correlations between intrinsic motivation, parental physical activity, and parental support are presented in Supplementary Table S2. Parental support was positively associated with interest/ enjoyment (*p* < 0.001), perceived competence (*p* < 0.001), perceived choice (*p* < 0.001), and value/ usefulness (*p* = 0.005 for parental co-activity; *p* < 0.001 for parental concern), while negatively associated with pressure/ tension (*p* = 0.007 for parental co-activity; *p* < 0.001 for parental concern). The associations between parental physical activity and items of intrinsic motivation were insignificant.

### 3.2. Change in physical activity during follow-up

At follow-up, 272 (11.2%) children and adolescents were physically active. Of the initially active participants (*n* = 1,120), 168 (15.0%) reported sufficient physical activity participation at follow-up (active maintainers). Of the initially inactive participants (*n* = 1,304), only 104 (8.0%) were active at follow-up (increasers). Patterns of physical activity change during follow-up are presented in Table 2.

**Table 2:** Change of physical activity during follow-up.

		Follow-up	
Baseline		Active (*n* = 272, 11.2%)	Inactive (*n* = 2,152, 88.8%)
	Active (*n* = 1,120, 46.2%)	168 (15.0%)	952 (85.0%)
	Inactive (*n* = 1,304, 53.8%)	104 (8.0%)	1,200 (92.0%)

### 3.3. Predictors of physical activity change

The longitudinal effects of predictors on physical activity maintenance are presented in Table 3. In the unadjusted model, physical activity was significantly associated with all the items of intrinsic motivation (OR = 1.375 for interest/enjoyment, OR = 1.303 for perceived competence, OR = 0.859 for pressure/tension, OR = 1.404 for perceived choice, and OR = 1.498 for value/usefulness). Parental physical activity (OR = 1.013) and parental concern (OR = 2.106 for always care) were also associated with physical activities. Similar results were observed after controlling for demographic variables. In the multivariable model, physical activity remained significantly associated with intrinsic motivation (OR = 1.341 for perceived choice) and parental concern (OR = 1.922 for always care).

**Table 3:** Predictors of physical activity maintenance among the initially active participants.

	OR_u_ (95% CI)	*p* value	OR_a_ (95%CI)	*p* value	OR_m_ (95%CI)	*p* value
Intrinsic motivation						
Interest/Enjoyment	1.375 (1.206–1.569)	<0.001	1.396 (1.220–1.597)	<0.001		
Perceived Competence	1.303 (1.160–1.465)	<0.001	1.311 (1.164–1.475)	<0.001		
Pressure/Tension	0.859 (0.764–0.965)	0.011	0.854 (0.759–0.961)	0.009		
Perceived Choice	1.404 (1.231–1.602)	<0.001	1.403 (1.229–1.601)	<0.001	1.341 (1.173–1.533)	<0.001
Value/Usefulness	1.498 (1.196–1.876)	<0.001	1.493 (1.191–1.871)	<0.001		
Parental physical activity^*^	1.013 (1.003–1.023)	0.014	1.012 (1.001–1.023)	0.026	1.011 (1.000–1.022)	0.053
Parental concern^*^						
Occasionally	Ref		Ref		Ref	
Frequently	1.411 (0.880–2.261)	0.126	1.445 (0.896–2.331)	0.132	1.400 (0.862–2.272)	0.174
Always	2.106 (1.344–3.301)	0.001	2.227 (1.407–3.523)	<0.001	1.922 (1.204–3.068)	0.006
Parental coactivity^*^						
Never	Ref		Ref			
1 ~ 4 times per month	0.814 (0.526–1.259)	0.354	0.829 (0.530–1.296)	0.410		
>1 time per week	1.104 (0.711–1.715)	0.659	1.181 (0.746–1.870)	0.478		

^*^Participants with missing data on parental physical activity, parental level of concern about their child’ s physical activity, and parental frequency of coactivity with their child (*n* = 57) were excluded from analyses.

OR_u_: Odd ratios for univariable analysis.

OR_a_: Odd ratios adjusted for sociodemographic variables (sex, grade, father’s educational level, and family annual income).

OR_m_: Odd ratios for the final multivariable analysis.

CI: Confidence Interval.

The longitudinal effects of predictors on physical activity increase are presented in Table 4. In the unadjusted model, physical activity was significantly predicted by interest/enjoyment (OR = 1.215) and perceived choice (OR = 1.210). For parental variables, only parental co-activity significantly predicted physical activity (OR = 2.143 for >1 time per week). These associations were not altered by adjustment for demographic variables. Results from the multivariable model showed that perceived interest/enjoyment (OR = 1.193) and parental co-activity (OR = 1.995 for >1 time per week) were significantly associated with physical activity participation at follow-up.

**Table 4:** Predictors of physical activity increase among the initially inactive participants.

	OR_u_ (95%CI)	*p* value	OR_a_ (95%CI)	p value	OR_m_ (95%CI)	*p* value
Intrinsic motivation						
Interest/Enjoyment	1.215 (1.075–1.372)	0.002	1.119 (1.058–1.360)	0.005	1.193 (1.046–1.362)	0.009
Perceived Competence	1.097 (0.976–1.232)	0.121	1.078 (0.956–1.214)	0.221		
Pressure/Tension	0.955 (0.849–1.075)	0.448	0.957 (0.849–1.079)	0.473		
Perceived Choice	1.210 (1.074–1.363)	0.002	1.217 (1.078–1.373)	0.001		
Value/Usefulness	1.008 (0.879–1.155)	0.991	1.009 (0.877–1.157)	0.919		
Parental physical activity^*^	1.012 (0.996–1.027)	0.142	1.009 (0.993–1.025)	0.287		
Parental concern^*^						
Occasionally	Ref		Ref		Ref	
Frequently	1.581 (0.947–2.641)	0.080	1.625 (0.969–2.726)	0.065		
Always	1.501 (0.887–2.542)	0.130	1.597 (0.993–2.726)	0.086		
Parental coactivity^*^						
Never	Ref		Ref		Ref	
1 ~ 4 times per month	1.360 (0.778–2.379)	0.281	1.415 (0.801–2.502)	0.232	1.361 (0.768–2.410)	0.291
>1 time per week	2.143 (1.199–3.830)	0.010	2.215 (1.222–4.013)	0.009	1.995 (1.095–3.633)	0.024

^*^ Participants with missing data on parental physical activity, parental level of concern about their child’ s physical activity, and parental frequency of coactivity with their child (*n* = 65) were excluded from analyses.

OR_u_: Odd ratios for univariable analysis.

OR_a_: Odd ratios adjusted for sociodemographic variables (sex, grade, father’s educational level, and family annual income).

OR_m_: Odd ratios for the final multivariable analysis.

CI: Confidence Interval.

## 4. Discussion

This longitudinal study aimed to examine whether intrinsic motivation, parental physical activity, and parental support were associated with the dramatic change in physical activity during the COVID-19 pandemic using the SDT framework. In our study, physical activity levels decreased significantly over 3 months. Intrinsic motivation and parental support were found to have simultaneous effects on both physical activity maintenance and increase. The results encourage physical activity interventions targeting personal motivation or parent–child interaction in children and adolescents.

In this study, only 15.0% of the initially active children and adolescents maintained sufficient physical activity. Meanwhile, a small group (8.0%) of initially inactive participants reported sufficient physical activity at follow-up. The pronounced trend toward declining may be attributed to the study time. During the early phase of the COVID-19 pandemic, children and adolescents were unable to adequately maintain their normal physical activity patterns due to home confinement (29, 30). Consistent with a systematic review, a significant decrease in physical activity during this special period was observed in the present study (23). Our findings provide important evidence on the contributing factors to physical activity maintenance and increase when children and adolescents experience major life events, in our case, the COVID-19 pandemic.

According to the hypothetic model, personal motivation and parental factors significantly influence physical activity in children and adolescents (10, 31). In this study, intrinsic motivation emerged as a significant predictor for physical activity. Results of the multivariable models further demonstrated the significance of perceived choice and enjoyment in maintaining and increasing physical activity. In addition, parental factors could play different roles in physical activity change. We found that parental concern was related to maintained physical activity in the initially active children and adolescents, while parental co-activity was related to increased physical activity in the initially inactive children and adolescents.

As indicated by SDT, physical activity behavior is innately driven by intrinsic motivation. Some researchers suggested that while extrinsic motivation could support initial physical activity behavior, intrinsic motivation is necessary for long-term behavioral change (32). Consistent with this SDT-grounded tenet, this study demonstrated the positive effect of intrinsic motivation on maintained physical activity. Furthermore, results from the multivariable analyses extend the previous findings by revealing that perceived self-choice is the most critical facet of intrinsic motivation that contributes to physical activity maintenance. For children and adolescents who are already engaged in physical activity, providing autonomously supportive conditions is a promising avenue to acquire greater health benefits (33). In contrast, interest and enjoyment, the inherent component of SDT (34), played an integral role in increasing physical activity among the initially inactive participants in this study. As a positive affective experience, enjoyment provides immediate and tangible rewards for being active. Consequently, individuals with higher perceived enjoyment tend to engage in physical activity spontaneously without external reinforcement (35). Therefore, enjoyment-oriented interventions (e.g., gamification design) are needed to increase physical activity participation in children and adolescents (36, 37).

The present study also illustrated the positive effects of parental factors on physical activity over time. However, the significance of parental physical activity was completely nullified in the multivariable model. Inconsistent findings have been evidenced on the association between parent and child physical activity. A systematic review indicates that methodological differences between studies could partially explain the heterogeneity in the results, with stronger associations reported using objective measures of physical activity (21). Moreover, in recognition of the complex nature of physical activity change, the importance of parental physical activity may diminish when considered alongside other forms of parental influence (38). Our results correspond to a previous finding that sustained physical activity was predicted by parental support but not role modeling when examined simultaneously (39). Therefore, family-based interventions targeting parental physical activity might be insufficient, and more direct parent–child interactive strategies are needed.

Regarding parental support, we found that parental concern and co-activity could predict maintained and increased physical activity, respectively. Trost et al. suggest that different forms of parental support could impact their child’s physical activity indirectly through its influence on self-efficacy (40). Children and adolescents with insufficient physical activity tend to have low levels of self-efficacy as a result of perceived external barriers such as transportation or equipment (41). Parental co-activity increases children’s confidence in their ability to participate in physical activity *via* providing practical assistance, which in turn can lead to increased physical activity levels (42). However, for those already involved in physical activity, the influence of this tangible support becomes less influential due to their higher perception of self-efficacy (41). In contrast, intangible parental support may play a critical role in physical activity maintenance among active children and adolescents (17). The findings of our study indicate that family-based interventions should prioritize different constructs of parental support, contingent upon the varying physical activity levels of children and adolescents.

Our study is the first longitudinal study that provides a clearer insight into the key factors underlying the dramatic changes in physical activity levels during the COVID-19 pandemic. Today, the physical activity levels of children and adolescents remain low and consistently show a trend of decreasing with age. Our findings have important implications for maintaining a high level of physical activity during childhood and adolescence. In addition, children and adolescents’ lifestyle behaviors are especially vulnerable to some major life events, which may again lead to a dramatic short-term decrease in physical activity. In response to these life-changing events, the SDT-related elements, namely intrinsic motivation and parental support, can be utilized to reduce fluctuation in children and adolescents’ physical activity.

This study has several limitations. First, physical activity was investigated within a short period of 3 months. However, the impacts of dramatic lifestyle changes were particularly evidenced in this special study period. Second, because the second survey was conducted during home quarantine and school closure, information was only collected on out-of-school physical activity at follow-up. This special study time allowed us to gain a clearer understanding of the role of family-related factors on physical activity. Third, we relied on self-reported measures of physical activity, which is subject to potential reporting bias. Future studies may use objective measures, such as accelerometers, to track longitudinal physical activity in children and adolescents. Fourth, although the present study measured parental co-activity and parental concern, other important aspects of parental support (e.g., provision of encouragement or transportation) were not incorporated. Future research may consider investigating the influence of these supportive parental behaviors on physical activity changes in children and adolescents. Finally, the sample in the present study was limited to Shanghai. Therefore, the present finding should be generalized with caution.

## 5. Conclusion

This longitudinal study focused on the short-term physical activity change during the COVID-19 pandemic among children and adolescents. Our findings demonstrated the significant effects of intrinsic motivation and parental support on physical activity over time. Specifically, perceived self-choice and parental concern about their child’s physical activity predicted maintained physical activity among the initially active children and adolescents, while perceived enjoyment and parental co-activity with their child predicted increased physical activity among the initially inactive children and adolescents. Interventions should capitalize on intrinsic motivation and parental support to promote physical activity and confront the overall decreasing trend during adolescence.

## Data availability statement

The raw data supporting the conclusions of this article will be made available by the authors, without undue reservation.

## Ethics statement

The studies involving human participants were reviewed and approved by Ethics Committee of Shanghai Jiao Tong University School of Medicine. Written informed consent to participate in this study was provided by the participants' legal guardian/next of kin.

## Author contributions

MX: conceptualization, methodology, and funding acquisition. MX, YL, XG, and EZ: data curation. YL and FH: formal analysis. YL and XG: Writing – original draft. MX, YC, and HL: writing – review and editing. All authors contributed to the article and approved the submitted version.

## Funding

This study was funded by Shanghai Municipal Education Commission (grant number 2023-Sports, Health, Arts and Science Department 01-45), the National Natural Science Foundation of China (grant number 71804110), Shanghai Science and Technology Development Funds (grant number 21QA1405300), Science Foundation for new teachers of Shanghai Jiao Tong University School of Medicine (grant number 20×100040012), and Shanghai Municipal Health Commission (GW-10.1-XK07).

## Acknowledgments

We thank all the teachers and students’ parents involved in this study for their contributions and efforts in conducting the survey.

## Conflict of interest

The authors declare that the research was conducted in the absence of any commercial or financial relationships that could be construed as a potential conflict of interest.

## Publisher’s note

All claims expressed in this article are solely those of the authors and do not necessarily represent those of their affiliated organizations, or those of the publisher, the editors and the reviewers. Any product that may be evaluated in this article, or claim that may be made by its manufacturer, is not guaranteed or endorsed by the publisher.

## Supplementary material

The Supplementary material for this article can be found online at: https://www.frontiersin.org/articles/10.3389/fpubh.2023.1175439/full#supplementary-material
