# Supplementary material for: Physical activity maintenance and increase in Chinese children and adolescents: the role of intrinsic motivation and parental support
Source: Front Public Health. 2023 Jul 31;11:1175439. doi: 10.3389/fpubh.2023.1175439 (PMC10424444; doi:10.3389/fpubh.2023.1175439)
Supplement: Supplementary file 1 [file Table_1.DOCX]

Supplementary Table 1. Comparison between the excluded and retained participants.

| Characteristics | Total (n=2424) | Excluded (n=122) | Retained (n=2302) | p value |
| --- | --- | --- | --- | --- |
| Gender |  |  |  |  |
| Male | 1241 (51.2%) | 73 (59.8%) | 1168 (50.7%) | 0.050 |
| Female | 1183 (48.8%) | 49 (40.2%) | 1134 (49.3%) |  |
| Age | 11.1±2.67 | 11.0±2.68 | 11.1±2.67 | 0.587 |
| Grade |  |  |  |  |
| 1-3 | 573 (23.6%) | 27 (22.1%) | 546 (23.7%) | 0.411 |
| 4-6 | 858 (35.4%) | 50 (41.0%) | 808 (35.1%) |  |
| 7-9 | 993 (41.0%) | 45 (36.9%) | 948 (41.2%) |  |

Data are presented as mean±standard deviation or median (Q1—Q3 quartiles) for continuous variables and n (%) for categorical variable

Supplementary Table 2. Correlations among intrinsic motivation, parental physical activity, and parental support

|  | Interest/ Enjoyment | Perceived Competence | Pressure/ Tension | Perceived Choice | Value/ Usefulness | Parental PA | Parental co-activity | Parental concern |
| --- | --- | --- | --- | --- | --- | --- | --- | --- |
| Interest/Enjoyment | — |  |  |  |  |  |  |  |
| Perceived Competence | 0.721*** | — |  |  |  |  |  |  |
| Pressure/Tension | -0.487*** | -0.464*** | — |  |  |  |  |  |
| Perceived Choice | 0.566*** | 0.627*** | 0.536*** | — |  |  |  |  |
| Value/Usefulness | 0.360*** | 0.466*** | -0.479*** | 0.506*** | — |  |  |  |
| Parental PA | -0.016 | -0.007 | -0.007 | 0.006 | 0.007 | — |  |  |
| Parental co-activity | 0.168*** | 0.159*** | -0.076** | 0.135*** | 0.080** | 0.127*** | — |  |
| Parental concern | 0.162*** | 0.181*** | -0.135*** | 0.153*** | 0.105*** | 0.012 | 0.300*** | — |

*p < 0.05; **p < 0.005;***p < 0.001
